# Vitamin D Deficiency and Replacement Challenges in Type 1 Gastric Neuroendocrine Tumors: A Comparative Study

**DOI:** 10.3390/nu18020281

**Published:** 2026-01-15

**Authors:** Elio Benevento, Michele Coletta, Alessia Liccardi, Roberto Minotta, Gianfranco Di Iasi, Massimo Di Nola, Annamaria Colao, Roberta Modica

**Affiliations:** 1 Endocrinology, Diabetology and Andrology Unit, Department of Clinical Medicine and Surgery, University of Naples Federico II, 80131 Naples, Italy; michele99coletta@gmail.com (M.C.); alessia.liccardi@yahoo.com (A.L.); robertominotta@gmail.com (R.M.); gianfrancodiiasi@gmail.com (G.D.I.); massimodinola96@gmail.com (M.D.N.); colao@unina.it (A.C.); 2 UNESCO Chair, Education for Health and Sustainable Development, University of Naples Federico II, 80131 Naples, Italy

**Keywords:** gastric neuroendocrine tumors, vitamin D, entero-pancreatic neuroendocrine tumor

## Abstract

**Background/Objectives**: Type 1 gastric neuroendocrine tumors (gNET) arise in the setting of autoimmune chronic atrophic gastritis and secondary hypergastrinemia. Vitamin D deficiency (VDD) has been associated with bone impairment and adverse outcomes in patients with neuroendocrine tumor (NET); however, data specifically addressing gNET remain limited. This study aimed to evaluate vitamin D status, supplementation requirements, and bone involvement in patients with type 1 gNET compared with those with entero-pancreatic NET (EP-NET). **Methods**: This retrospective study included patients with type 1 gNET followed at a tertiary referral center between 2010 and 2025 and an age- and sex-matched EP-NET cohort. VDD prevalence, time and dose required for normalization, supplementation formulations, bone status, and dietary habits were analyzed. **Results**: Twenty-six patients were included (thirteen gNET and thirteen EP-NET). VDD was significantly more prevalent in the gNET group compared with the EP-NET group (92.3% vs. 46.2%, *p* = 0.03, OR: 14). gNET required significantly higher daily cholecalciferol doses (3198.9 ± 1629 vs. 1580 ± 1121 IU/day, *p* = 0.008) and more frequently required multiple supplementation formulations (38.5% vs. 0%, *p* = 0.04). Multivariable linear regression analysis restricted to VDD patients confirmed that gNET was independently associated with higher daily cholecalciferol dose requirements (*p* = 0.037). Bone impairment, defined as osteoporosis or osteopenia, was significantly more common in the gNET group (61.5% vs. 15.4%, *p* = 0.04, OR: 8.8). Dietary adherence did not differ between groups. **Conclusions**: Type 1 gNET show a higher burden of VDD, increased vitamin D supplementation requirements, and a higher prevalence of bone impairment compared with EP-NET, irrespective of dietary habits. These findings suggest disease-specific mechanisms and support the need for tailored management in these patients.

## 1. Introduction

Gastric neuroendocrine tumors (gNET) represent a heterogeneous group of neoplasms, with type 1 gNET accounting for approximately 70–80% of cases and typically arising in the setting of chronic atrophic gastritis, due to autoimmune gastritis or chronic gastric infection [1]. The pathogenesis of type 1 gNET involves parietal cell loss, leading to achlorhydria and compensatory hypergastrinemia, which drives enterochromaffin-like cell hyperplasia and subsequent tumor development [2].

Clinically, type 1 gNET are usually small, indolent lesions with a low metastasic potential. Nevertheless, they require careful surveillance and management tailored to tumor size, grade, and stage [1]. Chronic atrophic gastritis, particularly when severe and corpus-restricted, impairs intrinsic factor production, resulting in altered absorption of several nutrients, including vitamin B12 and fat-soluble vitamins such as vitamin D [3].

Vitamin D plays a multifaceted role in patients with gastroenteropancreatic neuroendocrine tumors (GEP-NET) and pulmonary carcinoids. Observational studies suggest that lower serum 25-hydroxy(25OH)-vitamin D levels correlate with more aggressive disease and poorer outcomes, while vitamin D supplementation may be associated with improved progression-free and overall survival, although available evidence remains limited and a causal pathophysiological link has not yet been established [4,5,6]. Despite these observations, data specifically addressing vitamin D metabolism in patients with type 1 gNET are scarce. In particular, little is known about the prevalence and severity of vitamin D deficiency in this population, the dosage and duration of supplementation required to achieve biochemical normalization, and the potential need for alternative cholecalciferol formulations. This issue is clinically relevant, as cholecalciferol is available in multiple formulations—including oral drops, capsules, tablets, orodispersible films, and intermittent high-dose preparations—which may differ in efficacy depending on gastrointestinal absorption and patient-related factors [7].

Against this background, the present study aimed to compare patients with type 1 gNET and age- and sex-matched patients with entero-pancreatic neuroendocrine tumors (EP-NET) in terms of the following: prevalence of 25OH-vitamin D deficiency (VDD); daily cholecalciferol dose and time required to achieve vitamin D normalization; and number of vitamin D formulations needed. By addressing these aspects, this paper sought to clarify whether type 1 gNET represents a distinct clinical condition with specific vulnerabilities in vitamin D metabolism and bone health.

## 2. Materials and Methods

### 2.1. Study Population

The study population included adult patients aged 45–75 years with a confirmed diagnosis of type 1 gNET or EP-NET, referred to the Department of Endocrinology, Diabetology and Andrology of the University of Naples Federico II, European Neuroendocrine Tumor Society (ENETS) Center of Excellence, between January 2010 and April 2025. EP-NET patients were randomly selected and matched for age and sex to serve as a control group.

### 2.2. Inclusion and Exclusion Criteria

To ensure data homogeneity, patients with malabsorptive gastrointestinal diseases (e.g., celiac disease), history of malabsorptive gastrointestinal surgery (e.g., bariatric surgery or stoma creation), genetic syndromes, and impaired renal function were excluded. Specifically, patients with chronic kidney disease stage ≥ IIIb were not eligible for inclusion. Only patients with non-functioning NET were included. Patients receiving chronic high-dose vitamin D supplementation prior to the diagnosis of hypovitaminosis D were excluded. Occasional low-dose supplementation before diagnosis was allowed, reflecting routine clinical practice; however, baseline serum 25OH-vitamin D levels were measured at the time hypovitaminosis D was first documented.

### 2.3. Data Collection

Demographic, clinical, biochemical, and histological data, together with bone metabolism parameters, were retrospectively collected from medical records. Body mass index (BMI) was calculated as weight (kg) divided by height squared (m^2^), and obesity was defined according to standard criteria. Hypovitaminosis D was defined as serum 25OH-vitamin D levels < 20 ng/mL and severe deficiency as levels < 12 ng/mL, according to current guidelines [8]. All 25OH-vitamin D measurements were performed in accredited laboratories affiliated with the Italian National Health System, using chemiluminescent immunoassay (CLIA) throughout the entire study period (2010–2025). No changes in assay methodology occurred, ensuring consistency and comparability of results. Daily cholecalciferol doses were calculated by dividing the total administered dose over the treatment period by the number of days of supplementation. For intermittent regimens (e.g., monthly dosing), the total monthly dose was divided by 30 to obtain the equivalent IU/day. Mean doses are reported to allow comparability across patients. Osteoporosis or osteopenia were diagnosed based on dual-energy X-ray absorptiometry (DXA) and/or radiological assessment of vertebral morphology, as suggested by current guidelines [9]. NET grading was defined according to the Ki-67 index [10]. Follow-up was evaluated in months up to the last clinical visit.

### 2.4. Dietary Assessment

To exclude a potential influence of dietary habits on 25OH-vitamin D serum levels, adherence to the Mediterranean diet (MD) was assessed using the “Prevention with Mediterranean Diet (PREDIMED)” questionnaire [11]. The questionnaire was administered by telephone to all enrolled patients, who were asked to refer specifically to the period in which VDD was diagnosed. The PREDIMED questionnaire consists of 14 items, with a maximum achievable score of 14 points. Scores ≤ 5 indicate low adherence, scores between 6 and 9 indicate medium adherence, and scores ≥ 10 indicate good adherence to the MD [11].

### 2.5. Statistical Analysis

Statistical analysis was performed using SPSS version 22.0 for Windows (SPSS Inc., Chicago, IL, USA). Normality of continuous variables was assessed using the Shapiro–Wilk test and visual inspection of Q–Q plots. Variables with approximately normal distribution were compared using Student’s *t*-test, whereas the Mann–Whitney U test was applied to non-normally distributed variables. Categorical variables were compared using Fisher’s exact test due to the small sample size. All statistical tests were two-sided, and a *p* value < 0.05 was considered statistically significant. In linear regression models, β coefficients are reported and represent the estimated change in the dependent variable for a one-unit increase in the predictor, while holding all other variables constant. Effect sizes are provided to improve clinical interpretability and are expressed as odds ratios (ORs) with corresponding 95% confidence intervals (CIs) for categorical outcomes and as β coefficients with 95% CIs for continuous outcomes.

## 3. Results

**Figure 1:**
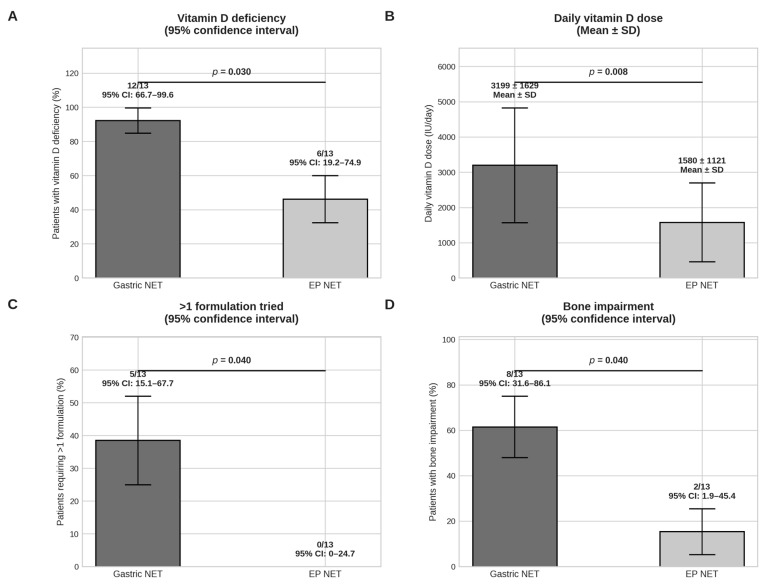
Comparison of vitamin D deficiency, supplementation requirements, and bone impairment between patients with gastric neuroendocrine tumors (gNET) and entero-pancreatic neuroendocrine tumors (EP-NET). (**A**) Prevalence of vitamin D deficiency (VDD) expressed as percentage of affected patients (*n* = 13 per group), with 95% confidence intervals (CIs). (**B**) Daily cholecalciferol dose required to achieve normalization of serum 25-hydroxy-vitamin D levels, expressed as mean ± standard deviation (IU/day). (**C**) Proportion of patients requiring more than one cholecalciferol formulation to normalize serum 25-hydroxy-vitamin D levels, expressed as percentage with 95% CIs (*n* = 13 per group). (**D**) Prevalence of bone impairment (osteoporosis or osteopenia), expressed as percentage with 95% CIs (*n* = 13 per group). Exact *p*-values are reported within each panel. Error bars indicate standard deviation for continuous variables and binomial 95% confidence intervals for categorical variables.

A total of 13 patients with type 1 gNET were enrolled (4 males and 9 females, mean age at diagnosis 59.76 ± 7.6 years old). An equal number of 13 age- and sex-matched EP-NET patients were randomly selected as a control group after verification of inclusion and exclusion criteria. Among gNET patients, 10/13 (76.9%) had autoimmune chronic atrophic gastritis, with positivity of anti-parietal cell antibodies in 9/10 (90%) and of anti-intrinsic factor antibodies in 1/10 (10%), while 3/13 (23.1%) had chronic gastric infections that resulted in atrophic gastritis. Among EP-NET patients, tumor localization was pancreatic in nine cases (69.2%), ileal in two (15.4%), duodenal in one (7.7%), and rectal in one (7.7%). Obesity was diagnosed in eight gNET and in six EP-NET patients (*p* = 0.70, Table 1). BMI was slightly higher in the gNET cohort but without statistical significance (30.85 ± 4.22 kg/m^2^ vs. 28.21 ± 3.03 kg/m^2^, *p* = 0.08). Eleven gNET and nine EP-NET patients were G1 (84.6% vs. 69.2%, *p* = 0.65); the other patients were G2 (*p* = 0.65). Other autoimmune diseases were diagnosed in six gNET (four with Hashimoto’s thyroiditis and two with rheumatoid arthritis) and in four EP-NET patients (three with Hashimoto’s thyroiditis and one with Graves’ disease); none had type 1 diabetes mellitus. VDD was significantly more prevalent in patients with gNET compared with EP-NET patients (92.3% vs. 46.2%, *p* = 0.03; Table 1 and Figure 1A), corresponding to an OR of 14.0 (95% CI: 1.4–141). Patients with gNET required significantly higher daily doses of vitamin D supplementation to achieve normalization compared with EP-NET patients (3198.9 ± 1629.1 IU/day vs. 1580 ± 1120.8 IU/day, *p* = 0.008, Table 1 and Figure 1B). The time required to normalize 25OH-vitamin D levels did not differ significantly between groups, although it tended to be longer in gNET patients (27.7 ± 20.54 vs. 14.6 ± 18.4, *p* = 0.12). A significantly higher proportion of gNET patients required more than one vitamin D formulation to normalize serum levels (38.5% vs. 0%, *p* = 0.04, Table 1 and Figure 1C). According to time of commercial introduction, orodispersible films were the last formulation to be tried. Interestingly, 3/13 (23.1%) of the gNET cohort needed orodispersible cholecalciferol films to achieve normal 25OH-vitamin D levels, while no patients had to try them in the EP-NET cohort. All patients underwent radiological assessment for bone status after VDD diagnosis. Bone impairment, defined as osteoporosis or osteopenia, was significantly more common in the gNET group compared with the EP-NET group (61.5% vs. 15.4%, *p* = 0.04, Table 1 and Figure 1D), corresponding to an OR of 8.8 (95% CI: 1.35–57.43). Mean PREDIMED scores were comparable between gNET and EP-NET patients (9.25 ± 0.87 vs. 8.85 ± 1.21, respectively; *p* = 0.33). Similarly, the distribution of Mediterranean diet adherence categories did not differ between groups, with comparable proportion of patients showing good and medium adherence to the MD (*p* = 1.00, Table 1). No patients in either group exhibited low adherence. Due to the small sample size and imbalance in vitamin D deficiency prevalence, multivariable logistic regression models showed evidence of quasi-complete separation. Therefore, parsimonious models were preferred, and results were interpreted cautiously. In multivariable linear regression analysis restricted to patients with vitamin D deficiency, gNET status was independently associated with higher daily cholecalciferol dose requirements (β = +1463 IU/day, 95% CI: 100–2826 IU/day, *p* = 0.037), whereas BMI and autoimmune comorbidities were not significant predictors.

## 4. Discussion

In this retrospective study, a significantly higher prevalence of VDD was observed in patients with type 1 gNET compared with those with EP-NET (*p* = 0.03), despite similar demographic and clinical characteristics. In addition to statistical significance (*p* = 0.03), effect size analysis revealed a strong association between gNET status and VDD, with a substantially increased OR, although the confidence interval was wide due to the limited sample size. These findings indicate that type 1 gNET represents a distinct clinical condition with an intrinsically higher susceptibility to vitamin D deficiency, without implying direct causation.

Beyond prevalence, important differences emerged in vitamin D replacement requirements. Patients with gNET required significantly higher daily doses of cholecalciferol and more frequently needed changes in vitamin D formulation to achieve biochemical normalization (*p* = 0.008 and *p* = 0.04, respectively). Multivariable linear regression analysis restricted to patients with VDD confirmed that gNET status was independently associated with higher daily dose requirements (β = +1463 IU/day, *p* = 0.037), whereas BMI and concomitant autoimmune diseases were not significant predictors. Although logistic regression for VDD was limited by quasi-separation due to the small sample size, the strong association between gNET status and vitamin D deficiency persisted across univariate and adjusted analyses, supporting the robustness of the observed effect and the hypothesis of disease-specific mechanisms affecting vitamin D absorption or metabolism, while still precluding causal inference.

Importantly, bone impairment, defined as osteoporosis or osteopenia, was significantly more prevalent in the gNET group, with a markedly increased odds ratio compared with EP-NET patients (*p* = 0.04, OR = 8.8). While causality cannot be established, this association suggests that altered vitamin D status in gNET patients may be linked to a higher burden of skeletal involvement, highlighting potential clinical implications that merit further investigation.

Several potential confounding factors that could influence vitamin D status were carefully evaluated. Importantly, sex, age, BMI, tumor grading, and the presence of concomitant autoimmune diseases did not differ significantly between groups. Although a trend toward higher BMI values was observed in gNET patients, this difference did not reach statistical significance (*p* = 0.08) and is unlikely to fully account for the marked discrepancy in VDD prevalence and supplementation requirements, particularly given the persistence of gNET status as an independent predictor in multivariable analysis.

Dietary habits were also assessed using the PREDIMED questionnaire, administered retrospectively to assess adherence to the MD during the period in which VDD was diagnosed. Former observational studies suggested that higher adherence to the MD is independently associated with higher circulating vitamin D levels, principally because of high consumption of fish and olive oil, foods that contribute to dietary vitamin D intake and may enhance its absorption [12,13]. Mean PREDIMED scores and adherence categories were comparable between groups, with no patient showing low adherence. Although the retrospective administration of the questionnaire represents a limitation, these findings argue against a major dietary contribution to the observed differences in vitamin D status.

**Figure 2:**
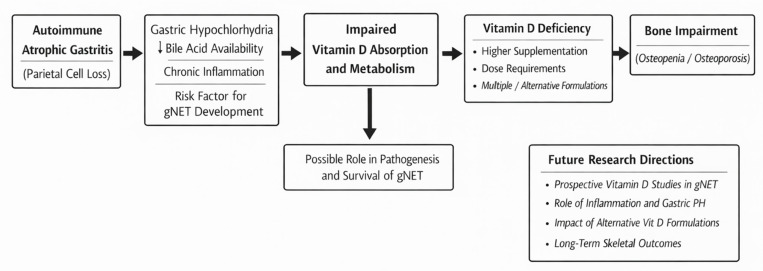
Conceptual framework linking type 1 gastric neuroendocrine tumor (gNET), vitamin D deficiency, and bone impairment. Autoimmune chronic atrophic gastritis leads to gastric hypochlorhydria, altered bile acid physiology, and chronic inflammation, which may impair vitamin D absorption and metabolism. These mechanisms contribute to a higher prevalence of vitamin D deficiency, increased supplementation requirements, and the need for multiple or alternative formulations in patients with type 1 gNET, ultimately resulting in a higher burden of bone impairment. The scheme also highlights potential areas for future research.

The higher prevalence and severity of vitamin D deficiency in gNET patients may instead be related to disease-specific mechanisms. Type 1 gNET typically arise in the context of autoimmune chronic atrophic gastritis, which was present in the majority of the gNET patients in our cohort [2]. Gastric atrophy, hypochlorhydria, and associated autoimmune processes may impair vitamin D absorption or metabolism, potentially explaining both the higher prevalence of deficiency and the reduced responsiveness to standard oral supplementation [14]. In fact, hypochlorhydria related to gastric atrophy impair fat digestion and micelle formation, while autoimmune-mediated chronic inflammation may further disrupt intestinal vitamin D absorption, hepatic activation, and peripheral vitamin D signaling [8,15]. Moreover, altered bile acid secretion and subtle pancreatic exocrine dysfunction—both described in chronic gastric and autoimmune conditions—may further compromise absorption of fat-soluble vitamins (Figure 2) [15]. The need for higher doses and alternative formulations, including orodispersible cholecalciferol films, further supports the hypothesis of altered gastrointestinal handling of vitamin D in these patients [16]. Novel formulations such as sublingual or buccal vitamin D preparations may offer advantages in patients with malabsorption by bypassing the gastrointestinal tract and delivering vitamin D directly into the systemic circulation [8,17,18,19,20]. A randomized crossover trial demonstrated that vitamin D buccal spray produced significantly higher serum 25OH-vitamin D concentrations compared to soft gelatin capsules in both healthy subjects and patients with malabsorption syndrome [18]. These findings suggest that alternative delivery methods should be considered in gNET patients who fail to respond adequately to conventional oral supplementation. Presented data are consistent with emerging evidence suggesting a complex interaction between vitamin D metabolism, autoimmunity, and NET [5]. Vitamin D has been shown to exert antiproliferative, pro-differentiative, and immunomodulatory effects through the vitamin D receptor, which is expressed in various neuroendocrine tissues [4,6]. Previous studies have reported an association between low vitamin D levels and worse outcomes in NET patients, although data remain limited and heterogeneous [5,6]. A large retrospective study of 172 patients with neuroendocrine neoplasms found that VDD was highly prevalent (68%) and was associated with a higher Ki-67 proliferation index and disease progression [19]. Similarly, another study of 75 patients with GEP-NET demonstrated that VDD was associated with a higher tumor grade (G2 vs. G1) and progressive disease, with patients exhibiting VDD or severe VDD having shorter progression-free survival [5]. Importantly, vitamin D supplementation has been associated with improved overall survival in NET patients, suggesting a potential therapeutic role beyond skeletal health [16]. The mechanisms underlying the association between VDD and NET aggressiveness are likely multifactorial. Vitamin D signaling has been shown to regulate key pathways involved in tumorigenesis, including the p53/AMP-activated protein kinase/Mammalian Target of Rapamycin (mTOR) pathway, which controls autophagy and cell proliferation [21]. In gastric cancer cells, vitamin D has been demonstrated to promote autophagy and apoptosis while inhibiting cell cycle progression through activation of this pathway [21]. Additionally, vitamin D modulates the expression of microRNAs that regulate cancer stem cell proliferation and epithelial–mesenchymal transition, processes that are critical for tumor metastasis and drug resistance [22]. In this context, this study adds novel evidence highlighting a distinct vulnerability of gNET patients to vitamin D deficiency and bone impairment, compared with EP-NET patients. The observation of a significantly higher prevalence of bone impairment in the gNET cohort further reinforces the clinical importance of our findings, suggesting that alterations in vitamin D metabolism in this specific population may translate into meaningful long-term consequences for bone health [16]. In a large cross-sectional study including 401 postmenopausal women, it was demonstrated that atrophic gastritis was independently associated with 89% increased odds of osteoporosis after adjustment for multiple confounding factors, including age and body mass index [23]. These findings provide a biological and clinical framework that is consistent with the higher burden of bone impairment observed in gNET patients in our cohort.

### Study Limitations and Strengths

Several limitations of this study should be acknowledged. First, the retrospective design precludes the assessment of causal relationships, while the relatively small sample size reduces statistical power, particularly for multivariable analyses. Therefore, non-significant results should be interpreted cautiously and do not exclude clinically relevant effects. Second, certain variables that may influence vitamin D status and bone health—such as season of sampling and sun exposure, but also some medication—were not available for statistical analysis. Moreover, the lack of detailed inflammatory markers, gastric pH measurements, bile acid profiling, and pancreatic exocrine function assessment limits the possibility of a deeper mechanistic interpretation. In addition, the administration of the PREDIMED after the diagnosis of hypovitaminosis D may have introduced recall bias, and adherence to home-based vitamin D supplementation could not be objectively assessed.

Despite these limitations, the study also has notable strengths. These include strict age- and sex-matching, the overall homogeneity of clinical characteristics between groups, a comprehensive evaluation of potential confounding factors, and the integration of effect size analyses alongside conventional *p*-values. Together, these methodological strengths enhance the robustness and clinical relevance of the findings.

## 5. Conclusions

Patients with type 1 gNET appear to be at increased risk of VDD, require higher supplementation doses, and experience a higher prevalence of bone impairment compared with EP-NET patients. These differences are not explained by demographic or dietary-related factors, suggesting disease-specific mechanisms related to gastric autoimmunity and atrophic gastritis. Thus, a careful evaluation of vitamin D metabolism and of bone health should be routinely considered in patients with type 1 gNET. Although limited by its retrospective design and relatively small sample size, this study provides novel insights into the metabolic and skeletal vulnerability of patients with type 1 gNET. Prospective, larger-scale studies are warranted to confirm these observations, clarify the underlying pathophysiological mechanisms, and define evidence-based guidelines for vitamin D monitoring and replacement tailored to this specific NET subgroup.

## Figures and Tables

**Table 1:** Population characteristics.

Variable	Gastric NET	EP-NET	Effect Size (95% CI)	*p*-Value (Test)
Population characteristics				
Number of patients	13	13	-	1.00 (Fisher)
Sex distribution (M:F)	4:9	4:9	-	1.00 (Fisher)
Age (years; mean ± SD)	59.76 ± 7.6	58.4 ± 8.3	-	1.00 (*t*-test)
Follow-up (months; mean ± SD)	89.00 ± 52.53	71.23 ± 62.80	-	0.42 (*t*-test)
BMI (kg/m^2^; mean ± SD)	30.85 ± 4.22	28.21 ± 3.03	-	0.08 *(t*-test)
Diagnosis of obesity	8/13 (61.5%)	6/13 (46.1%)	-	0.70 (Fisher)
eGFR (mL/min/1.73 m^2^)	78.4 ± 10.1	76.8 ± 8.9	-	0.90 (*t*-test)
PTH (pg/mL)	81.5 ± 12.6	78.8 ± 14.0	-	0.61 (*t*-test)
Calcium blood level corrected for albumin (mg/dL)	9.2 ± 0.4	9.1 ± 0.2	-	0.48 (*t*-test)
Grading G1	11/13 (84.6%)	9/13 (69.2%)	-	0.65 (Fisher)
Grading G2	2/13 (15.4%)	4/13 (30.8%)	-	0.65 (Fisher)
Autoimmune disease different from chronic atrophic gastritis	6/13 (46.2%)	4/13 (30.8%)	-	0.69 (Fisher)
Vitamin D status				
Diagnosis of VDD	12/13 (92.3%)	6/13 (46.2%)	OR: 14.0 (1.4–141)	0.03 (Fisher)
Diagnosis of severe VDD	3/13 (23.1%)	2/13 (15.4%)	OR: 1.65 (0.21–12.9)	1.00 (Fisher)
25OH-vitamin D blood levels at VDD diagnosis (ng/mL)	14.04 ± 3.30	13.32 ± 3.80	-	0.60 (*t*-test)
Treatment				
Months to 25OH-vitamin D normalization (mean ± SD)	27.7 ± 20.54	14.6 ± 18.4	-	0.12 (*t*-test)
Daily cholecalciferol dose (IU/day)	3198.9 ± 1629.1	1580 ± 1120.8	β: +1463 (100–2826)	0.008 (*t*-test)
>1 formulation tried	5/13 (38.5%)	0/13 (0%)	OR: 17.9 (0.9–∞) *	0.04 (Fisher)
25-OH-vitamin D normalization with orodispersible films	3/13 (23.1%)	0/13 (0%)	OR: 8.0 (0.4–∞) *	0.22 (Fisher)
Bone outcomes				
Osteoporosis	4/13 (30.8%)	2/13 (15.4%)	OR: 2.44 (0.35–17.0)	0.64 (Fisher)
Osteopenia	4/13 (30.8%)	0/13 (0%)	OR: 12.0 (0.6–∞) *	0.09 (Fisher)
Bone impairment (osteoporosis + osteopenia)	8/13 (61.5%)	2/13 (15.4%)	OR: 8.8 (1.35–57.43)	0.04 (Fisher)
Diet				
Score to PREDIMED test (mean ± SD)	9.25 ± 0.87	8.85 ± 1.21	-	0.33 (*t*-test)
Good adherence to MD	5/13 (38.5%)	4/13 (30.8%)	OR: 1.41 (0.28–7.04)	1.00 (Fisher)
Medium adherence to MD	8/13 (61.5%)	9/13 (69.2%)	-	1.00 (Fisher)
Low adherence to MD	0/13	0/13	-	1.00 (Fisher)

Legend: CI: Confidence Interval, M: Males, F: Females, SD: standard deviation, BMI: Body Mass Index; eGFR: Estimated Glomerular Filtration Rate, PTH: Parathyroid Hormone, VDD: Vitamin D Deficiency, OR: Odds Ratio, β: β Coefficient For Linear Regression Model, MD: Mediterranean Diet, PREDIMED: Prevention with Mediterranean Diet Questionnaire. * Exact OR reported due to zero cells; confidence intervals are wide because of small sample size.

## Data Availability

The data presented in this study are not publicly available due to ethical and privacy restrictions, as they contain sensitive clinical information. Data supporting the findings of this study are available from the corresponding author upon reasonable request and with permission of the local ethics committee.

## Abbreviations

The following abbreviations are used in this manuscript:

BMI	Body mass index
CI	Confidence interval
CLIA	Chemiluminescent immunoassay
DXA	Dual-energy X-ray absorptiometry
eGFR	Estimated glomerular filtration rate
ENETS	European Neuroendocrine Tumor Society
EP-NET	Entero-pancreatic NET
GEP-NET	Gastroenteropancreatic neuroendocrine tumor
gNET	Gastric neuroendocrine tumor
MD	Mediterranean diet
mTOR	Mammalian target of rapamycin
NET	Neuroendocrine tumor
OR	Odds ratio
PREDIMED	Prevention with Mediterranean diet
PTH	Parathyroid hormone
VDD	Vitamin D deficiency
